# Cecal Adenoma Presenting as a Large Bowel Intussusception in an Adult

**DOI:** 10.7759/cureus.17680

**Published:** 2021-09-03

**Authors:** Nicole M Reyes, Keith P Blankenship, Michael A Elrod

**Affiliations:** 1 Department of Surgery, Grandview Medical Center, Dayton, USA

**Keywords:** adult intussusception, colonic intussusception, preoperative reduction, cecal adenoma, en bloc resection

## Abstract

An adult intussusception is a relatively rare entity and is more commonly confined to the small bowel when it is found. The majority of the colonic intussusceptions in adults are caused by malignant neoplasms. Here we present the case of a 65-year-old female with a cecal adenoma as the lead point causing intussusception all the way to the splenic flexure. Controversy still exists regarding optimal treatment strategies, specifically the question of if it is safe or not to perform preoperative reduction prior to surgical resection in adult large bowel intussusceptions.

## Introduction

Intussusception was first described by Paul Barbette in 1674, when a proximal segment of the bowel (termed intussusceptum), invaginates or telescopes into the adjacent distal portion of the intestine (termed the intussuscipiens) [1]. The most common cause of intestinal obstruction in children, intussusception is a rare entity in adults accounting for only 1-5% of all intestinal obstructions in adults, and only 0.003-0.02% of all adult hospital admissions [2,3]. In contrast to children, the majority of adult intussusceptions have a conclusive etiology in about 70-90% of the cases. Of the colonic intussusceptions, up to two-thirds are likely to be of malignant etiology [4]. For decades, there has still remained controversy regarding optimal treatment strategy for these rare processes. Herein, we present the case of a 65-year-old female with a long segment colonic intussusception secondary to a benign cecal adenoma. This case report was previously presented as a poster at the 2021 Dayton Area Graduate Medical Education Community 22nd Annual Virginia C. Wood Resident Research Forum on May 6, 2021. 

## Case presentation

**Figure 1:**
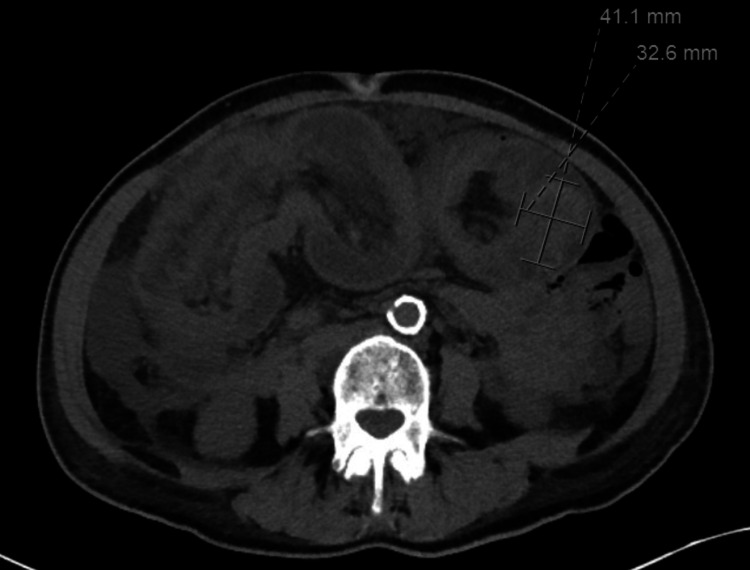
Axial slice of CT of the abdomen/pelvis demonstrating a large colonic intussusception in the mid-abdomen with extensive wall thickening and edema. Shown also is possible lead point at the distal portion of the invaginated bowl concerning for neoplasm.

**Figure 2:**
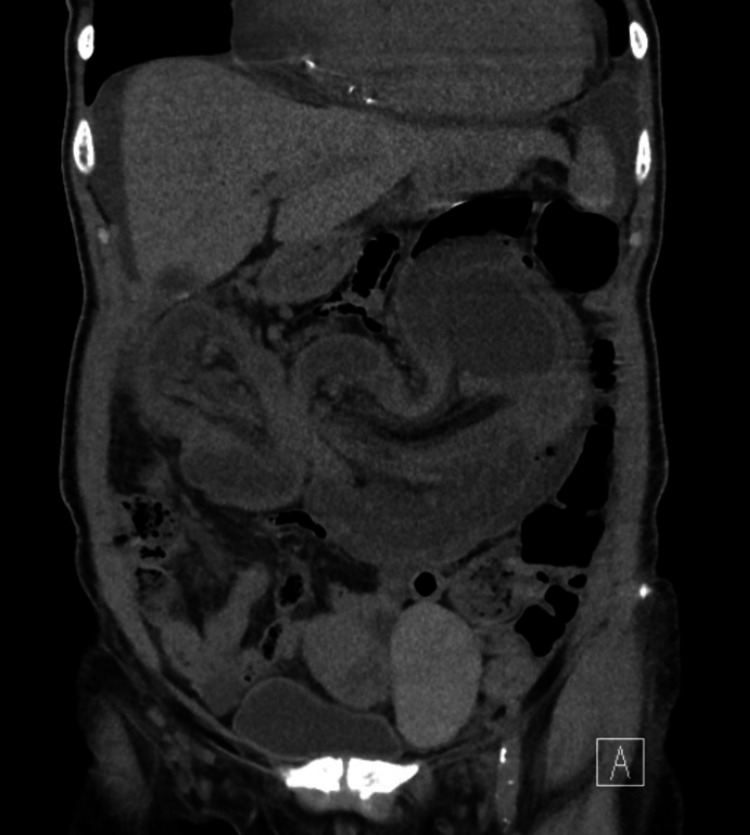
Coronal slice of the CT of the abdomen/pelvis demonstrating a large colonic intussusception in the mid-abdomen with extensive wall thickening and edema.

A 65-year-old female presented to the emergency department with the chief complaint of intermittent, periumbilical abdominal pain that had been ongoing for about one year. She reported associated, intermittent diarrhea over the past year as well. She had one episode of bright red blood per rectum two days prior, for which she presented to the emergency department. Work-up at that time was unremarkable and the patient was discharged home with instructions to follow up with the gastroenterology department. The patient had a medical history significant for congestive heart failure, hypertension, and uterine fibroids. Her surgical history included a myomectomy with no previous colonoscopies in the past. She denied any significant family history or the use of tobacco or alcohol. Upon evaluation in the emergency department, patient’s vitals appeared stable; she was afebrile and tolerating room air. Laboratory tests drawn were remarkable for a mildly elevated white blood cell count at 12.8 K/uL and a potassium of 2.5 mmol/L. On physical exam, patient was non-toxic appearing with complete resolution of her abdominal pain. Her abdomen was soft, non-distended, and non-tender, although there was a palpable mass located in her left upper quadrant. CT scan of her abdomen and pelvis revealed a large colocolonic intussusception in the mid-abdomen with extensive wall thickening/edema. It also revealed a hyperattenuating nodular opacity at the distal portion of the invaginated bowel, possibly representing a lead point/neoplasm as shown in Figure 1 and Figure 2.

**Figure 3:**
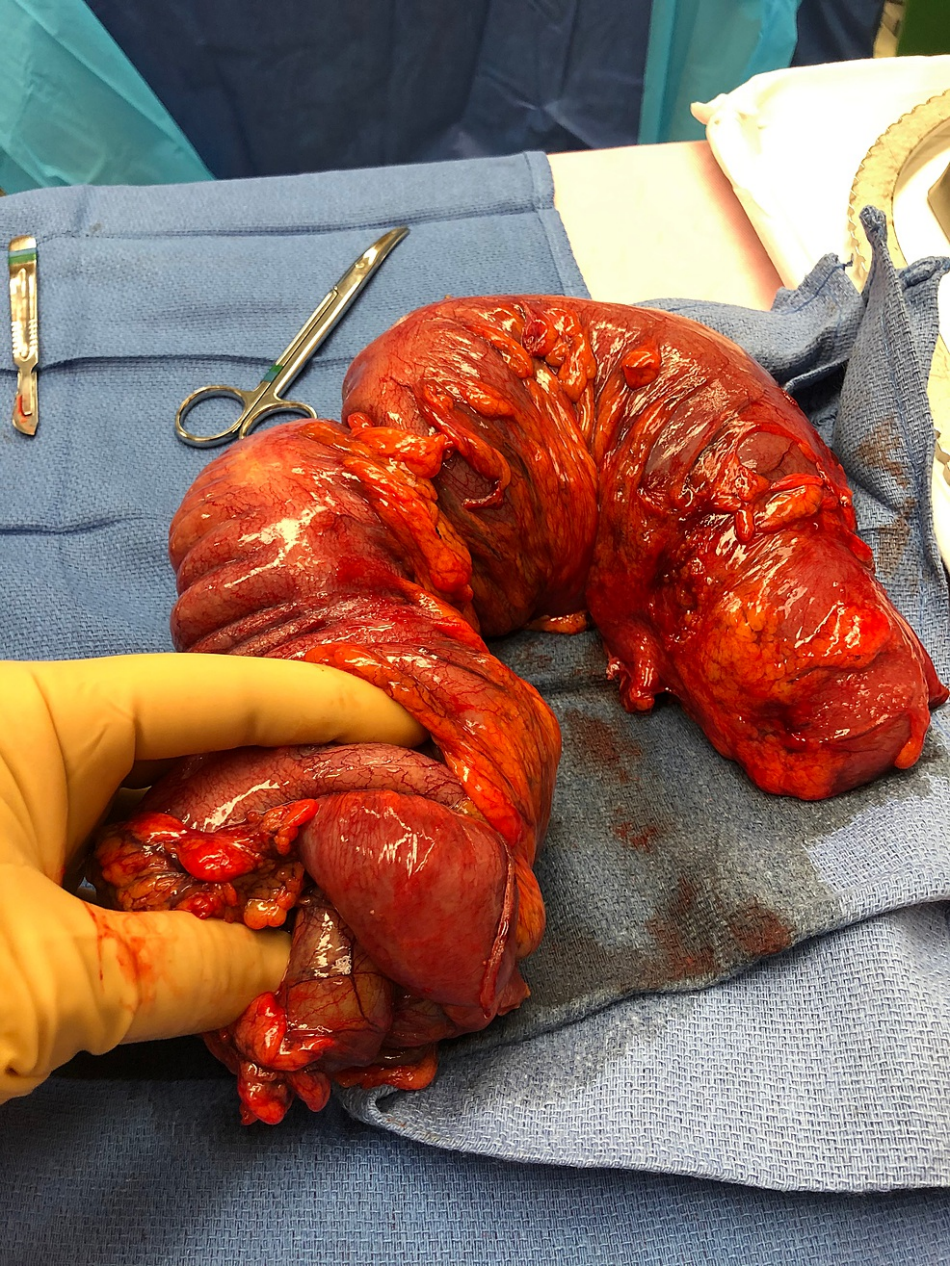
Intraoperative photograph of the en bloc resected specimen. The proximal staple line at the ileum can be seen at the bottom of the picture.

**Figure 4:**
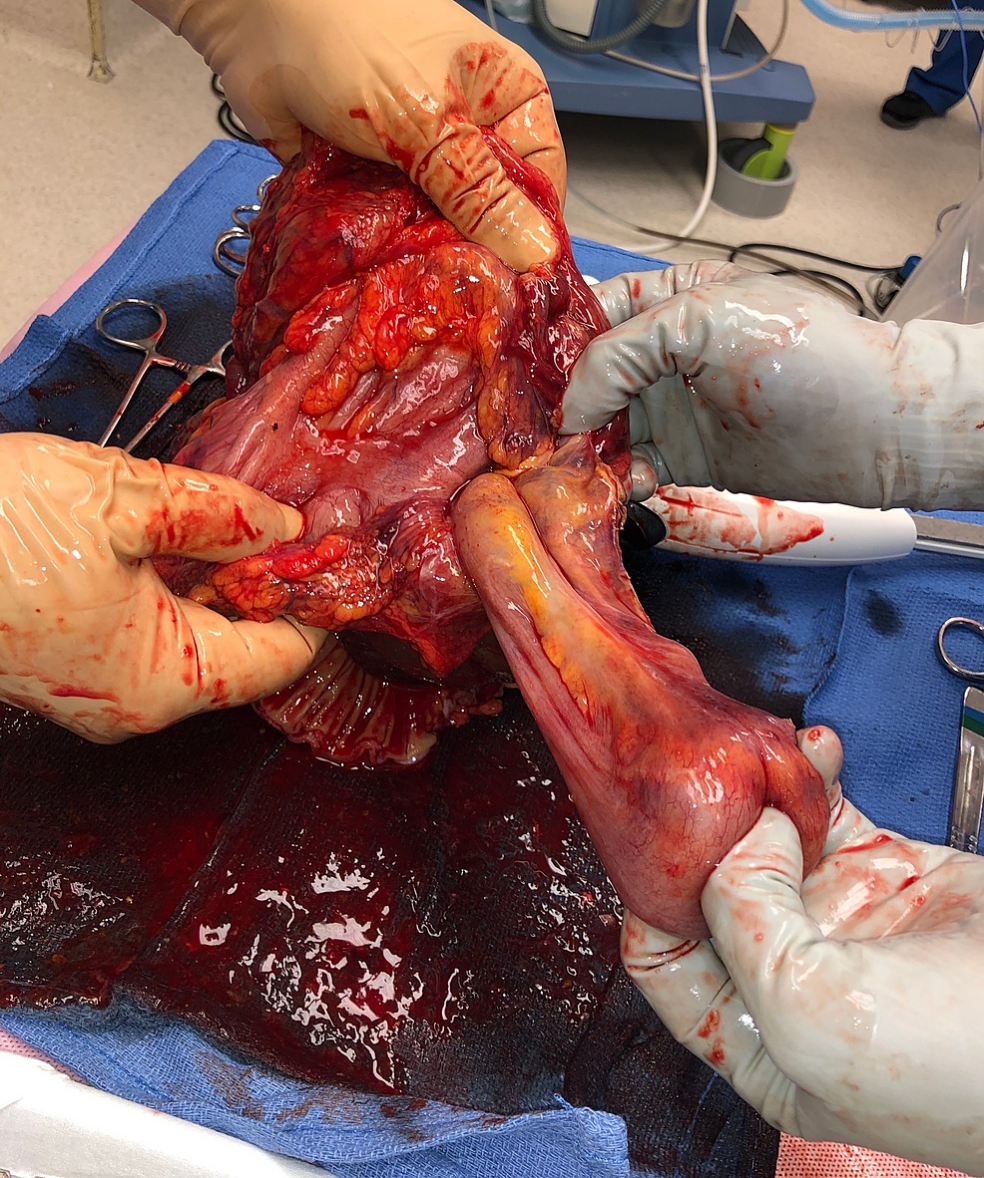
Intraoperative photograph of the intussusceptum being manually reduced on the back table, showing the bowel to appear viable.

**Figure 5:**
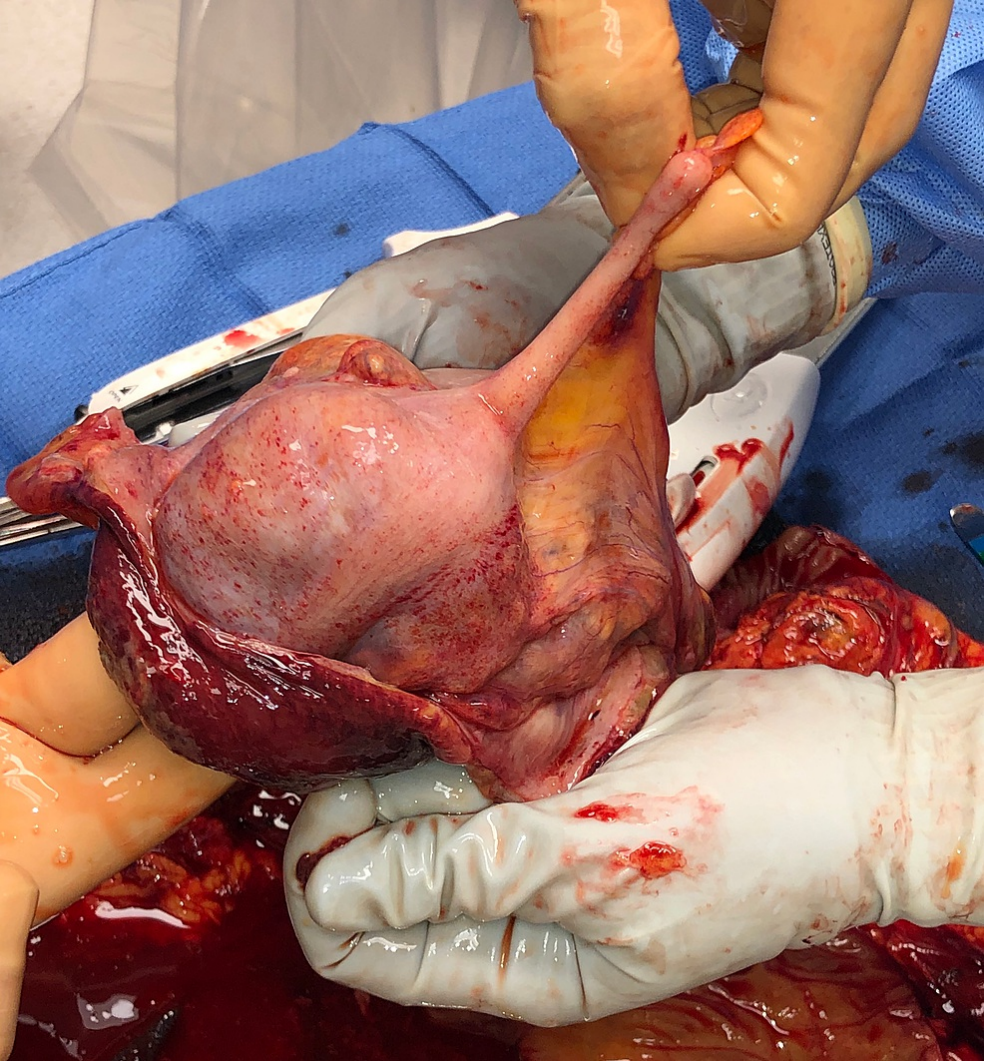
Intraoperative photograph of the visceral peritoneal surface of the cecum and appendix appearing viable.

**Figure 6:**
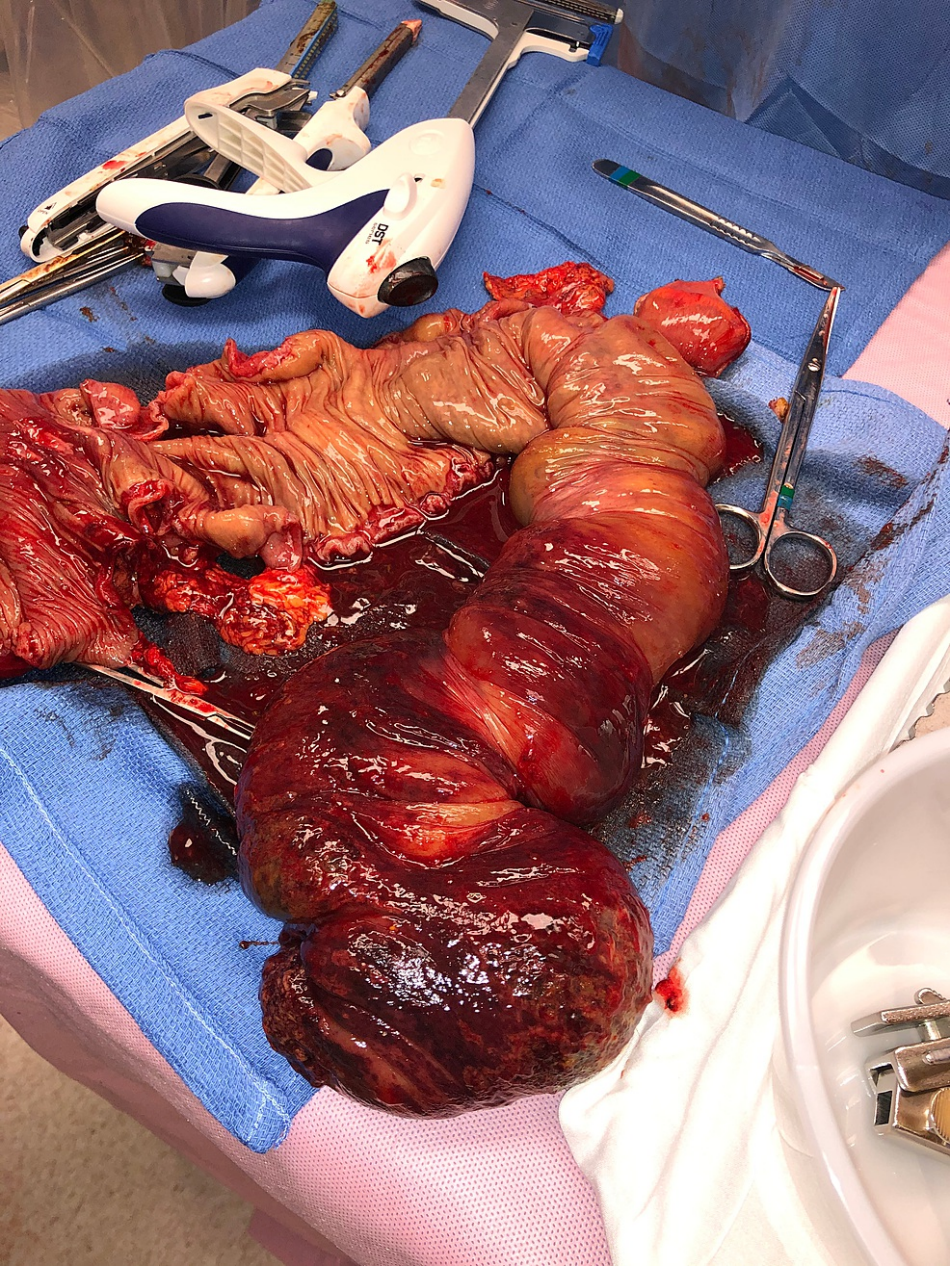
Intraoperative photograph of the entire specimen opened up revealing grossly mucosal ischemic changes of the cecum and ascending colon.

**Figure 7:**
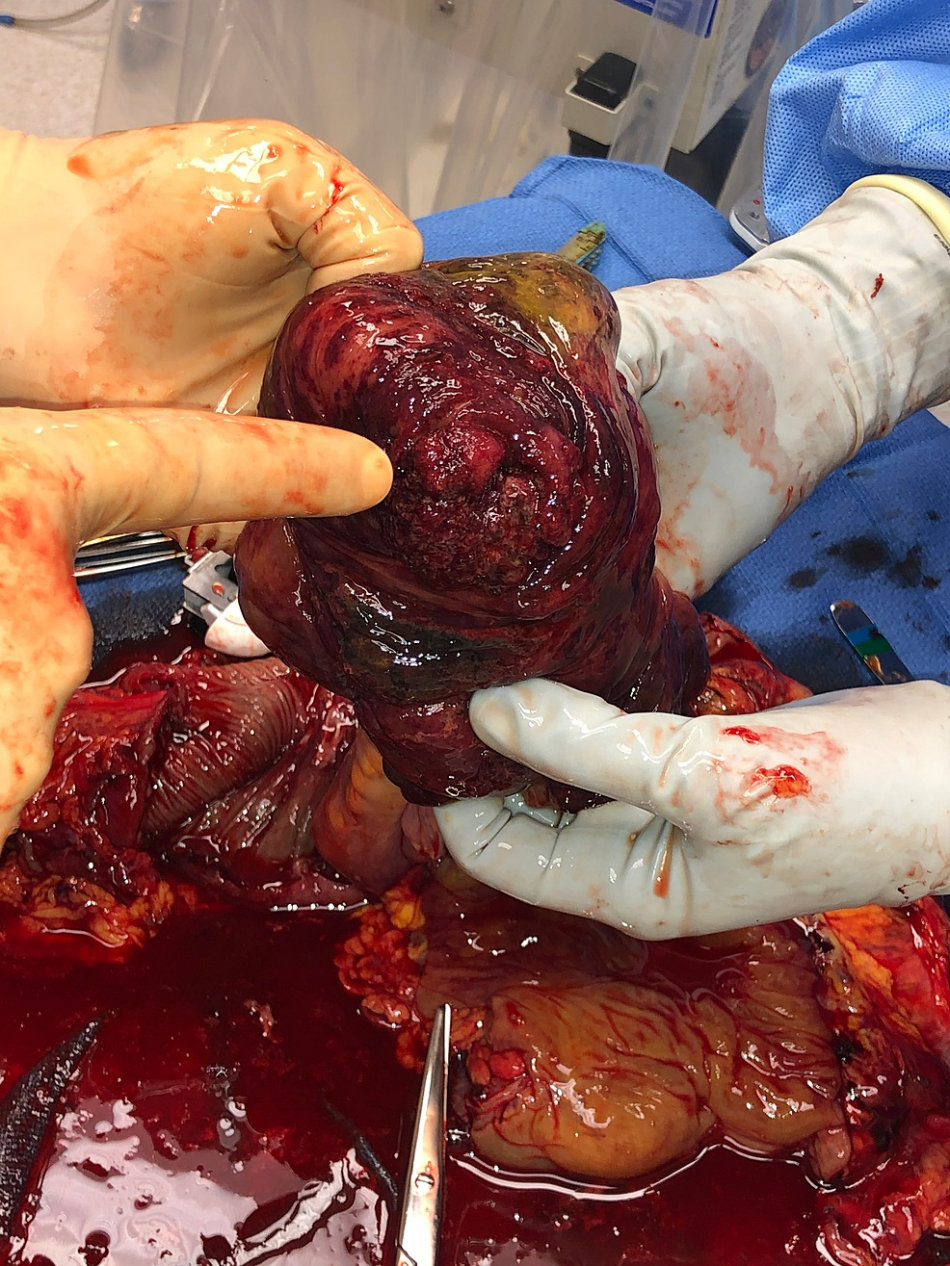
Intraoperative photograph of the cecal mass located at the appendiceal orifice.

Based on imaging findings and her suspected diagnosis of intussusception, patient was urgently taken to the operating room for exploratory laparotomy. Intraoperatively, the patient was discovered to have a large colonic intussusception, which appeared to invovle the cecum as the lead point, intussuscepting to the splenic flexure. The intussuscipiens did appear grossly viable; however, manual reduction was not attempted due to the long length of the intussusceptum. Patient subsequently underwent an extended right hemicolectomy with mobilization of the splenic flexure and primary anastomosis. Once the specimen was removed from the patient, it was manually reduced on the back table. At a glance, the intussusceptum did appear to be viable (Figure 3, Figure 4, Figure 5). However, once the specimen was opened up, the mucosal layer of the cecum and ascending colon did reveal grossly ischemic changes (Figure 6). A large cecal mass was also discovered and presumed to be the lead point (Figure 7).

The patient progressed appropriately postoperatively and was discharged home without complications. Pathology evaluation did reveal a 4.7cm cecal mass near the appendiceal orifice to be a tubulovillous adenoma, ischemic changes involving the cecum and ascending colon, and 25 lymph nodes negative for malignancy.

## Discussion

Intussusception in children is well documented as the most common cause of intestinal obstruction. Children often present with abdominal pain/cramping, vomiting, passage of bloody mucus, and at times with a palpable abdominal mass. In comparison to adults, the etiology of intussusception in children is usually idiopathic without an obvious lead point, therefore most pediatric surgeons are able to follow a protocol when it comes to treatment strategy. Hydrostatic reduction by enema using contrast material or air is the therapeutic procedure of choice [5]. About 74-79% are successfully reduced by hydrostatic or pneumatic enema. For those refractory to air enema attempts, a delayed repeat enema in a few hours has been proven safe. A third recurrence is usually an indication for operative intervention [6].

As previously stated, in contrast to children, adult intussusception is quite a rare disease process. Because of the infrequency of this phenomenon, there is still no optimal consensus regarding treatment strategy, which differs wildly from children. Traditionally, adult intussusceptions involving the colon have a malignant etiology until proven otherwise. Therefore, numerous studies have advocated for en bloc resection of the intussusception without attempt at reduction or manipulation [3,4].

Even though initial reduction might allow a more limited bowel resection and avoid emergency surgery [1], there still theoretically exist multiple risks for preoperative reduction or manipulation of the intussusception. There are theories of intraluminal seeding or venous embolization of malignant cells during operative manipulation as well as the risk of perforation and peritoneal soiling when there is bowel ischemia. In addition, there is the risk of anastomotic complications of the manipulated friable and edematous bowel tissue [3,7].

There have been recent reviews and case reports citing that the benefits of preoperative or intraoperative reduction of the intussusception outweigh the theoretical risks, especially if the etiology is of non-neoplastic origin [1,7,8]. However, it is very difficult to distinguish between intussusceptions that are neoplastic versus idiopathic. One recent article looked at trying to predict factors that would lean towards a malignant diagnosis so as to be more selective with surgical intervention. They found that chronic symptoms (greater than 14 days) and colonic intussusception were independent predictive factors of malignancy [9]. However, our patient displayed both of the predictive factors of malignancy, and yet the etiology turned out to be from an adenoma.

## Conclusions

As there are not enough high-quality data at this time showing successful outcomes with preoperative reduction or intraoperative manipulation, the question remains whether this is a safe or advisable option. Even though our patient’s etiology was of non-malignant origin, the intussusceptum showed evidence of diffuse mucosal ischemic changes. These areas of ischemia were not grossly obvious from the outside. If preoperative reduction or intraoperative manipulation were attempted in this case, it could have caused detrimental complications including perforation or even anastomotic complications secondary to the hidden mucosal edema and ischemia. Although the preservation of the bowel is important, the decision to pursue preoperative or intraoperative reduction of an adult intussusception involving the colon should be made cautiously. At this time the evidence still favors surgical intervention in adults with colonic intussusceptions. Even though there have been some cases of successful preoperative reduction in colonic intussusceptions, our case demonstrates that treatment strategy should likely be tailored to each individual patient.
